# The Royal Free Hospital

**Published:** 1908-03-14

**Authors:** 


### HOSPITAL ADMINISTRATION.

### CONSTRUCTION AND ECONOMICS.

### THE ROYAL FREE HOSPITAL.

The eightieth annual report, that for the year ending December 31 last, opens with congratulations upon the addition of two new operating theatres at the top of the Sussex wing. In thirty years the number of operations in this hospital has increased tenfold, and the cost of the new theatres, which have already been described in our columns (The Hospital, December 7, 1907, pp. 269-70), and of the necessary works amounted to upwards of £8,300.

The great need of the Royal Free Hospital at the moment is a new out-patient department, and any generous sympathiser with hospitals who would call on the Secretary and go into this matter with a view to defray the cost of an adequate site and to provide for the erection thereon of an up-to-date building would render an immeasurable service to the hospital and to the patients who flock to it in increasing numbers every year. Here is certainly an investment for the benevolent, which those who are wise will not delay investigating.

The finances of the hospital for the year are fairly satisfactory, as the total income from all sources, including legacies, amounted to £22,100, against a total expenditure of £25,600, which latter figure includes a sum of nearly £9,500 expended on structural and other improvements. When, however, the total ordinary income falls short of the total ordinary expenditure by nearly £7,000, as it did last year, it would appear as if the charter of free admission to all deserving cases, which has always been the leading feature of the Royal Free Hospital, had failed somehow recently to appeal with the same force it used to appeal to the giving public in all ranks of society. It seems to us that a special effort should be made to increase the revenue of the Royal Free Hospital by £7,000 a year. Why not organise an appeal under the highest auspices and set about the work during 1908? Such an appeal should be capable of being made effective from the fact that the increased cost of all London hospitals during the last few years has now probably attained its maximum, so that an additional £7,000 a year would make the Royal Free Hospital, in a financial sense, safe for many years to come.

In the course of 1907 (The Hospital, July 20, pp. 434-5) we brought out very clearly the excellent system of investigation into the circumstances of the patients which is carried on at this hospital under the supervision of two lady almoners and a corps of voluntary helpers. This system is so organised as to bring the work of the hospital into touch with the Charity Organisation Society, and all other agencies which can be usefully brought into co-operation, with a view to render the work efficient by bringing to bear every means best calculated to meet the requirements of every case that presents itself for treatment. These methods are well worthy of the attention of all hospital managers throughout the country. Another special feature of great importance in the work of this hospital is the annual census of in-patients, which ought to be adopted by every great hospital, because it enables the management to know exactly where the patients reside, and what portions of the community are specially benefited. This census shows the place of residence of all patients in the wards on a given Sunday in January. Of 153 patients last year, 42 resided in St. Pancras, 35 in Islington, 25 in Holborn, so that the neighbouring parishes supplied 102, or 66 per cent. of the total number of in-patients. Thirty-nine patients came from other London districts, and only 12 were received from the country.

We consider that these figures show that the work of the Royal Free Hospital is one which Londoners ought to specially support, because the larger proportion of its patients reside in the immediate neighbourhood of the hospital. At some hospitals there is a tendency to admit patients from the country to the exclusion of the poor who reside near its walls. This is a matter which should receive the earnest and close attention of the committees of management, for the first claim upon the accommodation in every London hospital must necessarily lie with the poor people who occupy tenements in the district surrounding each particular hospital. The experience at the Royal Free Hospital, as shown by the figures of the census for the last seven years, shows that this system tends to reduce the number of cases received from the country, and so places the maximum number of beds at the disposal of the poor recipient in the district which the hospital ought properly to serve.

